# Peer Review of “The Role of Animal-Assisted Therapy in Enhancing Patients’ Well-Being: Systematic Study of the Qualitative and Quantitative Evidence”

**DOI:** 10.2196/56047

**Published:** 2024-03-18

**Authors:** Anonymous

**Keywords:** animal-assisted therapy, pet therapy, outcome assessment, policies, systematic study

*This is a peer-review report submitted for the paper “The Role of Animal-Assisted Therapy in Enhancing Patients’ Well-Being: Systematic Study of the Qualitative and Quantitative Evidence.”*

## Round 1 Review

### General Comments

I enjoyed reading this paper [1]. In general, this is a well-written paper. There are some areas that could be clarified or expanded to improve the strength of the article. Due to the justification, the spacing of punctuation marks appears incorrect, and there are rare instances of double punctuation (periods). The use of American Psychological Association abbreviations at first use was not followed. At times, after providing an abbreviation, the full name is spelled out (eg, “AAT,” “PTSD”).

### Specific Comments

#### Major Comments

1. There does not appear to be a Table 2, but it is referenced in the text (page 7).

2. In the Discussion section on page 15, a reference is made to effect sizes in four outcome areas, yet no effect sizes were reviewed in the article.

3. Also in the Discussion on page 15, the word “power” is used: “The increased number of studies provided greater power in assessing variance heterogeneity and potential group differences.” Unless a specific power analysis was performed (if so, it should be discussed), the word “power” could be changed to “support” to reflect a review rather than an analysis.

Similarly, on page 16, the term “meta-analysis” is used. Unless a secondary analysis of pooled data was performed, the term “meta-analysis” should be changed to “analysis.” If a secondary pooled analysis was performed, that should be defined and described in the body of the paper.

4. Page 16, Society for Healthcare Epidemiology of America is noted as an organization of interest for animal-assisted therapy, as is Pet Partners. The authors may want to consider including the global organization called International Association of Human-Animal Interaction.

5. On page 17, the authors cite lack of blinding as a limitation and introduction of bias. I would be curious to know how the authors propose blinding in studies that involve interactions with animals. I strongly suggest this sentence be removed.

6. The work done by Hinic and others [2] was not a randomized controlled study as noted in Table 1. Please double-check that all studies listed are correctly labeled as randomized studies.

#### Minor Comments

7. Check punctuation for spacing.

8. Check all abbreviations and use abbreviations after first use is defined.

9. Check capitalizations in midsentence (page 4: “Dog”; page 7: “Unrepresentative”).

10. Page 6: “The articles should to be published in English.” Wrong tense—change to “were.”

11. Page 1: Three categories of interventions were provided in section 2.4. It would strengthen the paper to include definitions of these categories for the reader.
